# Comparative transcriptomics reveals the role of altered energy metabolism in the establishment of single-cell C_4_ photosynthesis in *Bienertia sinuspersici*

**DOI:** 10.3389/fpls.2023.1202521

**Published:** 2023-07-05

**Authors:** Sang-Yun Han, Woe-Yeon Kim, Jung Sun Kim, Inhwan Hwang

**Affiliations:** ^1^ Department of Life Sciences, Pohang University of Science and Technology, Pohang, Republic of Korea; ^2^ Division of Applied Life Science (BK21+) and Research Institute of Life Science, Institute of Agriculture and Life Sciences, Gyeongsang National University, Jinju, Republic of Korea; ^3^ Genomic Division, Department of Agricultural Bio-Resources, National Institute of Agricultural Sciences, Rural Development Administration, Jeonju, Republic of Korea

**Keywords:** *Bienertia sinuspersici*, *Suaeda aralocaspica*, *Amaranthus hypochondriacus*, single-cell C4 photosynthesis, transcriptome, dimorphic chloroplast, mitochondria, malate valve

## Abstract

Single-cell C_4_ photosynthesis (SCC_4_) in terrestrial plants without Kranz anatomy involves three steps: initial CO_2_ fixation in the cytosol, CO_2_ release in mitochondria, and a second CO_2_ fixation in central chloroplasts. Here, we investigated how the large number of mechanisms underlying these processes, which occur in three different compartments, are orchestrated in a coordinated manner to establish the C_4_ pathway in *Bienertia sinuspersici*, a SCC_4_ plant. Leaves were subjected to transcriptome analysis at three different developmental stages. Functional enrichment analysis revealed that SCC_4_ cycle genes are coexpressed with genes regulating cyclic electron flow and amino/organic acid metabolism, two key processes required for the production of energy molecules in C_3_ plants. Comparative gene expression profiling of *B. sinuspersici* and three other species (*Suaeda aralocaspica*, *Amaranthus hypochondriacus*, and *Arabidopsis thaliana*) showed that the direction of metabolic flux was determined via an alteration in energy supply in peripheral chloroplasts and mitochondria via regulation of gene expression in the direction of the C_4_ cycle. Based on these results, we propose that the redox homeostasis of energy molecules via energy metabolism regulation is key to the establishment of the SCC_4_ pathway in *B. sinuspersici*.

## Introduction

Plants are autotrophic organisms that acquire energy by photosynthesis, a process involving the conversion of carbon dioxide (CO_2_) into sugars using light energy (Edwards et al., 2010; Blätke & Bräutigam, 2019). The majority of plant species utilize C_3_ photosynthesis, in which CO_2_ is fixed into C_3_ compounds and eventually converted into sugars through the Calvin-Benson-Bassham (CBB) cycle (Edwards et al., 2004; Edwards et al., 2010). However, certain plant species exhibit C_4_ photosynthesis, which is characterized by the following steps: (1) CO_2_ capture by first fixing CO_2_ into C_4_ compounds; (2) shuttle of C_4_ compounds from one subcellular location to another; (3) release of CO_2_ from the C_4_ compound by malic enzyme or gluconeogenetic enzyme; (4) refixation of CO_2_ into C_3_ compound in the CBB cycle as in the C_3_ system; and (5) continuous production of carbon acceptor molecule for the first CO_2_ fixing (Bowes, 2010; Furbank, 2016; Schlüter et al., 2016; Blätke & Bräutigam, 2019). Terrestrial C_4_ photosynthetic plants can survive harsh weather conditions, suggesting that these plants have evolved mechanisms to adapt to their environment. C_4_ plants are more productive and have a higher carbon fixation efficiency compared to C_3_ plants, especially under high light and dry conditions (Edwards et al., 2004; Sage, 2004; Edwards et al., 2010; Hartzell et al., 2018; Blätke & Bräutigam, 2019).

C_4_ photosynthesis is categorized into different types by considering the enzymes responsible for releasing CO_2_ from the C_4_ compound: chloroplastic nicotinamide adenine dinucleotide phosphate-dependent malic enzyme (NADP-ME) and mitochondrial nicotinamide adenine dinucleotide-dependent malic enzyme (NAD-ME), pure form of phosphoenolpyruvate carboxykinase (PEPCK), and mixed form of either malic enzyme and PEPCK subtypes (Rao and Dixon, 2016; Blätke and Bräutigam, 2019). In addition, spatial separation between the first and second CO_2_ fixations is enabled via two mechanisms: Kranz anatomy, where the first and second CO_2_ fixation reactions occur in two different types of cells, mesophyll (M) and bundle sheath (BS) cells, respectively; and single-cell C_4_ (SCC_4_) system, where the two CO_2_ fixation reactions occur within a cell that possesses two different types of chloroplasts, named peripheral chloroplasts (PCs) and central chloroplasts (CCs), which are functionally equivalent to the chloroplasts in M and BS cells, respectively (Kadereit et al., 2003; Edwards et al., 2004; Offermann et al., 2015).

The development of Kranz anatomy in maize has been extensively studied (Hall et al., 1998; Rossini et al., 2001; Friso et al., 2010; Majeran et al., 2010; Slewinski et al., 2012; Wang et al., 2013; Fouracre et al., 2014; Tausta et al., 2014; Offermann et al., 2015). However, it remains largely unknown how dimorphic chloroplasts are developed within a single cell in terrestrial single-cell C_4_ (SCC_4_) plants such as *Bienertia sinuspersici* (‘Bienertia’ in short). Dimorphic chloroplasts are characterized by two different types of thylakoid stacking; PCs largely contain stromal thylakoids, whereas CCs possess stromal thylakoids as well as stacked grana (Offermann et al., 2015; Mai et al., 2019). In Bienertia, this dimorphic chloroplast feature underpins the establishment of dual electron flow in a chlorenchyma cell; linear electron flow (LEF) occurs in CCs, and cyclic electron flow (CEF) occurs in PCs. This dual system is responsible for the differential accumulation of two different types of energy molecules, adenosine triphosphate (ATP) and the reduced form of nicotinamide adenine dinucleotide phosphate (NADPH), in PCs and CCs, respectively.

In the SCC_4_ system, CO_2_ release from C_4_ compounds occurs within mitochondria (Edwards et al., 2004; von Caemmerer et al., 2014; Offermann et al., 2015). Although mitochondria-independent single-cell NADP-ME type C_4_ systems have been discovered in aquatic plants, no evidence of dimorphic chloroplasts has been found in these plants to date. Moreover, only NAD-ME type C_4_ photosynthesis has been reported in terrestrial SCC_4_ plants belonging to the family Amaranthaceae (Bowes, 2010; von Caemmerer et al., 2014; Han et al., 2020). Consequently, one key question arises regarding the relationship between CO_2_ release in the mitochondria-dependent NAD-ME type C_4_ system and dual electron flow in dimorphic chloroplasts in Bienertia. Recent proposals on the biochemical processes of SCC_4_ photosynthesis in Bienertia indicate that, similar to the NAD-ME type C_4_ system with Kranz anatomy, mitochondria need to be located adjacent to CCs to efficiently deliver CO_2_ to the Rubisco complex after decarboxylation by NAD-ME in mitochondria (Edwards et al., 2004; von Caemmerer et al., 2014). In addition, many metabolic processes are thought to be required for the proper functioning of the SCC_4_ system in Bienertia. Comparative analysis of the components of Kranz NAD-ME type C_4_ photosynthesis and C_3_ photosynthesis revealed that amino acid and organic acid metabolism-related genes of C_3_ species are recruited for C_4_ photosynthesis (Aubry et al., 2011; Igamberdiev & Eprintsev, 2016; Ludwig, 2016; Rao & Dixon, 2016; Rao et al., 2016; Khoshravesh et al., 2020; Borghi et al., 2022).

In this study, we analyzed the transcriptome of Bienertia leaves at the three developmental stages and performed comparative analysis of the transcriptome data with three other plant species. We focused on the expression patterns of energy metabolism- and C_4_ photosynthesis-related genes. In addition, we identified transporters involved in the C_4_ cycle, particularly those that transport metabolites between PCs and mitochondria. The results of transcriptome analysis revealed that genes related to the PC-localized CEF were coexpressed with those related to C_4_ photosynthesis in Bienertia. Moreover, biological function enrichment analysis showed that the production of C_4_ cycle metabolites was closely related to mitochondrial energy metabolism.

## Materials and methods

### Plant growth condition

*Bienertia sinuspersici* plants were grown in Magenta boxes containing Murashige and Skoog (MS) medium (1X MS salts, 10 mM MES [pH 5.8], 10 mM NaCl, 2% sucrose, 0.8% agar) in a chamber maintained at 16 h light/8 h dark cycle, an average of 75 mol quanta m^-2^ s^-1^ photon flux and 22°C temperature. Two-week-old plants were transferred to soil and grown in a greenhouse with an average of 150 mol quanta m^-2^ s^-1^ photon flux at 28°C. Plants were watered with 30 mM NaCl three times a week, and supplied with 1 g/L of 5.1-10-5 NPK fertilizer (BIO-NEX, South Korea) once a week. The leaf tissues were collected from 2–4-month-old plants grown in a greenhouse at 28°C to extract total RNA.

### Total RNA preparation

Leaf tissues were collected at three developmental stages (young, intermediate, and mature), as described previously (Koteyeva et al., 2016). Prior to processing the mature leaf tissues, only the top 1/3^rd^ portion of the leaves was used to minimize the inclusion of immature cells. Leaf tissues were ground in liquid nitrogen using a mortar and pestle. Total RNA was extracted using the CTAB-based method (Wang & Stegemann, 2010), and treated with the TURBO DNA-free™ kit (Invitrogen) to remove any contaminating DNA. The samples were resuspended in 50 µl DEPC-treated water and stored at -70°C for further analysis.

### Next generation sequencing and trimming of raw reads

Total RNA was used to generate cDNA libraries, which were subjected to 100 bp paired-end sequencing using the TruSeq Stranded mRNA Sample Preparation Kit (Illumina, CA, USA), according to the manufacturer’s instructions. The quality and quantity of cDNA libraries were evaluated using Agilent 2100 BioAnalyzer (Agilent, CA, USA) and the KAPA library quantification kit (Kapa Biosystems, MA, USA), respectively, according to the manufacturer’s instructions. Sequencing was carried out on the Illumina platform (Illumina, CA, USA) at Theragen Bio (Seongnam, South Korea). Clean reads were obtained with Trimmomatic (**Table S1**) (Bolger et al., 2014) using ILLUMINACLIP (Truseq3-PE:2:30:10), HEADCROP:5, TRAILING:20, AVQ:20, and MINLEN:36 parameters. Quality check of RNA-seq data was conducted by FastQC (Andrews, 2010).

### *De novo* transcriptome assembly and filtering of redundant transcripts

Transcripts obtained by sequencing all nine paired-end libraries were assembled using the *de novo* transcriptome assembler Trinity (Haas et al., 2013). Next, we quantified transcripts using RSEM (Li & Dewey, 2011). Redundant transcripts were filtered according to the following criteria: TPM < 1 and CD-HIT-EST = 0.95 (Li & Godzik, 2006; Haas et al., 2013; Bakkali et al., 2021). To obtain comprehensive information on the assembled transcriptomes, the sequences were annotated based on a similarity search against UniProt (The UniProt Consortium, 2021), NCBI nr (Sayers et al., 2022), and Araport11 (Cheng et al., 2017) using the BLASTx program (Camacho et al., 2009), based on a significant threshold E-value of ≤ 10^-10^. Raw Illumina reads were deposited in the Sequence Read Archive (SRA) database of NCBI under BioProject accession number PRJNA917470, and the assembled transcripts were deposited in NCBI under the accession number GKHT00000000.

### Differentially expressed genes analysis

**Figure 1:**
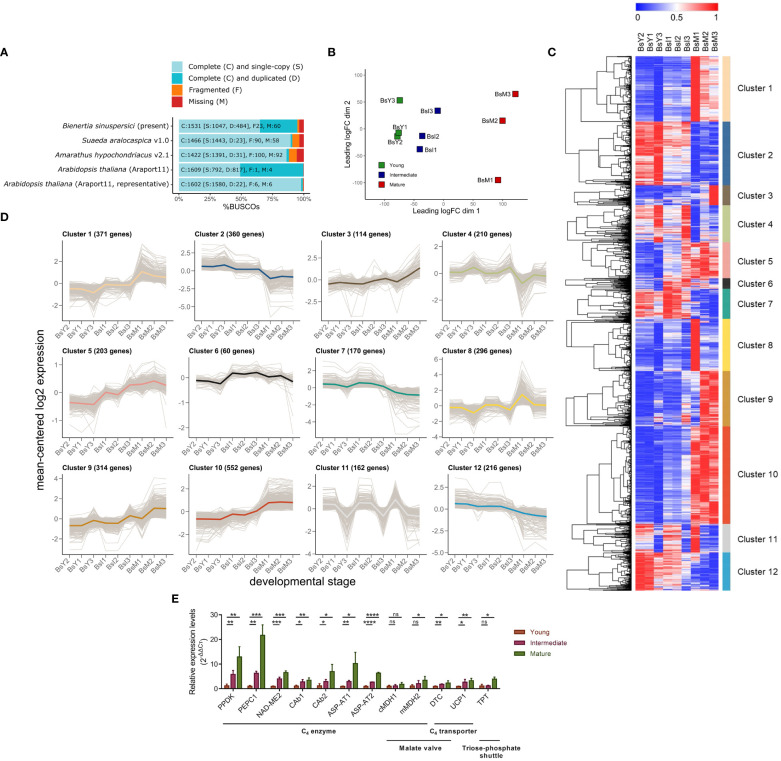
Transcriptional profiling of Bienertia leaf samples at three different developmental stages. **(A)** Benchmarking Universal Single-Copy Orthologs (BUSCO) transcriptome scores of Bienertia along with those of three comparative species, *Arabidopsis thaliana* (C_3_ type), *Amaranthus hypochondriacus* (NAD-ME/Kranz-C_4_ type), and *Suaeda aralocaspica* (NAD-ME/SCC_4_ type). **(B)** Classical multidimensional scaling (cMDS) plot. **(C)** Heatmap of differentially expressed genes (DEG) obtained by min-max normalized TPMTMM (blue [0, min] and red [1, max], respectively), dendrogram (trees), and K-means clustering data (colored bars). X-axis represents three biological replicates of RNA-seq samples taken from different stages of leaf development: Young leaf (BsY1, BsY2, BsY3), Intermediate leaf (BsI1, BsI2, BsI3), and Mature leaf (BsM1, BsM2, BsM3). Y-axis represents genes to visualize the variations in gene expression across different stages of leaf development. **(D)** Log2-transformed expression levels of genes in K-means clusters, with the gene number and mean-centered graph marked by colors assigned to each cluster. **(E)** Relative gene expression levels by qRT-PCR (n = 3, mean ± S.D.). The expression level was calculated by the 2^−ΔΔCt^ method. One-tailed Student’s T-tests were performed to determine significant differences between groups (Young vs. Intermediate or Young vs. Mature), and significance was denoted by asterisks (*p < 0.05; **p < 0.01; ***p < 0.001; ****p < 0.0001; ns, not significant). Gene abbreviations: *PPDK* (BS50819_c1_g1_i1), *PEPC1* (BS15679_c0_g1_i1), *NAD-ME2* (BS38916_c0_g1_i1), *CAb1* (BS301_c2_g1_i2), *CAb2* (BS7750_c0_g1_i10), *ASP-AT1* (BS6226_c0_g1_i3), *ASP-AT2* (BS1932_c0_g1_i7), *cMDH1* (BS95047_c2_g1_i1), *mMDH2* (BS6222_c0_g1_i2), *DTC* (BS12288_c0_g1_i2), *UCP1* (BS2112_c0_g1_i4), *TPT* (BS25442_c1_g1_i1).

Classical multidimensional scaling (cMDS) plot analysis was performed using the cmdscale function in R, with the average of the absolute value of Log2FC as leading pairwise distances of the gene expression between libraries (Leading logFC dim) (McCarthy et al., 2012; R Core Team, 2022). We performed DEG analysis using EdgeR and NOISeq programs (Robinson et al., 2010; Tarazona et al., 2015). To detect changes in the transcript levels and expression patterns of unigenes, young leaf tissues (Young) were used as the reference point. The determination of upregulation and downregulation in gene expression is based on the comparison of expression levels. When the expression level and proportion are higher in mature leaves compared to young leaves, it suggests upregulation. Conversely, when the expression level and proportion are higher in young leaves, it indicates downregulation in development. To identify key genes involved in the regulation of SCC_4_ photosynthesis in Bienertia, multifaceted comparisons were carried out (e.g., between the SCC_4_ species, between C_4_ plants and Kranz anatomy C_4_ plants, and between NAD-ME type C_4_ and C_3_ photosynthesis) using DEGs identified in the Young vs. Mature comparison. Aralocaspica (*Suaeda aralocaspica*) was used as another SCC_4_ species (Wang et al., 2019), and Amaranth (*Amaranthus hypochondriacus*) was used as a Kranz anatomy C_4_ species (Lightfoot et al., 2017). To compare the differences between the C_3_ and C_4_ systems, we used Aralocaspica and Amaranth as examples of NAD-ME type plants, and Arabidopsis (*Arabidopsis thaliana*) as an example of the C_3_ type plant. In the case of Arabidopsis, DEGs identified in the ‘Young cotyledon’ vs. ‘Adult cotyledon’ [CT-aCT] and ‘Cotyledon and shoot apical meristem’ vs. ‘Senescent leaf’ [CTSAM-SCLF] comparisons were used (Mergner et al., 2020). Additionally, the DEGs of Bienertia were compared with stress-related DEGs of Arabidopsis. These Arabidopsis DEGs were obtained from comparing ‘Non-stress’ [RC] vs. ‘Heat stress’ [RH] vs. ‘Salt/Heat stress’ [RSH] (Sewelam et al., 2020). This comparison was aimed to determine if any heat or salt stress-related regulations were involved in SCC_4_ cycle regulation. Comparative transcriptomic analysis was performed using OrthoVenn3 co-ortholog classification (Sun et al., 2023) and Araport11 and UniProt databases (Cheng et al., 2017; The UniProt Consortium, 2021). We used the UniProt database to describe gene annotation. When the Orthogroup-ID of OrthoVenn3 is identical among transcripts and the annotation of Araport11 is identical across species, we have determined that they are comparable (**Dataset S1**). Each dataset was prepared in a condition of both fragmented and missing scores of Benchmarking Universal Single-Copy Orthologs (BUSCO) to be < 100 at Embryophyta_odb10, Bowtie 2 re-mapping rate > 90%, and N50 value > 1.5 kb (**Figure 1A**; **Table S2**) (Langmead & Salzberg, 2012; Góngora-Castillo & Buell, 2013; Hölzer & Marz, 2019; Bakkali et al., 2021; Manni et al., 2021). The BUSCO score employs a collection of conserved orthologs to evaluate the completeness of transcriptome assemblies. It offers standardized metrics, facilitating unbiased quality comparisons across various studies and organisms (Manni et al., 2021). The normalization of quantified reads was carried out using the Transcript Per Million normalized by Trimmed Mean of the M values (TPMTMM) (de Vries et al., 2020). False discovery rate (FDR) < 0.05 (EdgeR) and probability (q) > 0.8 (NOISeq) were used as significance thresholds for Log2FC (McCarthy et al., 2012; Tarazona et al., 2015). The OmicsBox (https://www.biobam.com/omicsbox/) and Galaxy (https://usegalaxy.org/) platforms were used for all steps of RNA-seq data analysis, ranging from the trimming of RNA-seq reads to the analysis of DEGs.

The non-DEG transcripts, with very low expression levels, were filtered from the whole transcriptome for further analysis. As the minimum DEG between two TPMTMMs of a transcript, a ranking score (RS) of 20.1 was used based on Euclidean distance using fold change (FC) > 2 and absolute difference of expression level (D) > 20 (Tarazona et al., 2015). The RS is particularly noteworthy as it can identify significant differences in gene expression even in cases where either of the specified cut-off thresholds was not met. This capability allows it to capture extreme variations in gene expression levels.


$$RS\;=\;\sqrt{FC^{2}+D^{2}}$$

After filtering non-DEG transcripts, a heatmap of the DEGs was generated, and the DEGs were clustered by K-means clustering using the Morpheus software (https://software.broadinstitute.org/morpheus). To perform K-means clustering, the number of K-means clusters (K) ranging from 5 to 20 was used for DEGs. To perform hierarchical clustering, one minus Pearson correlation metric was selected to build the hierarchical tree for the complete linkage. Among the trials of K-means clustering, the most optimal clustering was obtained with K = 12. All information on the expression and annotation of *Bienertia sinuspersici* (Bienertia) genes, together with those of comparative plant species (*Suaeda aralocaspica* [Aralocaspica], *Amaranthus hypochondriacus* [Amaranth], and *Arabidopsis thaliana* [Arabidopsis]), is provided in the **Dataset S1** to **S7**.

### Biological function enrichment analysis

To conduct gene expression profiling, the ClusterProfiler package v4.0 of R (Wu et al., 2021) was used along with the Gene Ontology (GO) enrichment of K-means clusters. Metascape platform (https://metascape.org) was used to examine the relationship between gene expression patterns (K-means clusters) and functional GO, Kyoto Encyclopedia of Genes and Genomes (KEGG), and WikiPathways (WP) categories (Zhou et al., 2019) and to elucidate the developmental process of Bienertia. Cytoscape software v3.8.2 was used to conduct semantic network analysis and to generate Metascape plots (Shannon et al., 2003).

### Analysis of metabolic pathways and transporters

A model of central carbon metabolism of Bienertia was constructed based on pathways and chemical reactions in the BioCyc database (Karp et al., 2019) and Rhea database (Bansal et al., 2022). A transporter list was generated by combining both the Transporter Classification Database (TCDB) (Saier et al., 2021) and UniProt database (The UniProt Consortium, 2021) in such a way that TCDB ∪ UniProt [Keyword - Transmembrane (KW-0812) ∩ Keyword - Transport (KW-0813)]. The transporters were classified in four categories: Type I, electron transporters and proton pumps; Type II, protein transporters (related to proteins and peptides); Type III, macromolecule transporters (transport polymers, lipids, polyamines, wax, and vesicles); and Type IV, micromolecule transporters (translocate monomers, oligomers, metabolites, cations, anions, and extra small molecules). We then screened for putative SCC_4_ transporters, considering their substrate specificity, reaction mechanism, abundance and fold change.

### Gene expression analysis by quantitative real-time PCR

To validate the RNA-seq data of Bienertia, the expression profiles of genes were examined by qRT-PCR. The cDNAs of leaves at the young, intermediate, and mature stages of development were synthesized using the High-Capacity cDNA Reverse Transcription Kit (Applied Biosystems). Then, qRT-PCR was performed with the cDNA as template using PowerUp™ SYBR™ Green Master Mix (Applied Biosystems) under the following conditions: 95°C for 10 min, followed by 40 cycles of 95°C for 15 s and 60°C for 1 min. All reactions were performed in three biological replicates. We utilized *RPT6A* (BS4939_c0_g1_i1) and *SAND* (BS31299_c0_g1_i4), identified as non-DEGs in our transcriptome, as multiple reference genes for qRT-PCR data normalization (**Dataset S1**). The suitability of these reference genes was previously validated in other plant species (Czechowski et al., 2005; Chen et al., 2021), ensuring reliable normalization of our data. Primer sequences are listed in **Table S3**. Relative gene expression was calculated using the 2^−ΔΔCt^ method (Schmittgen & Livak, 2008).

## Results

### The expression pattern of genes shows a great deal of changes along with the development of leaf tissues

To elucidate C_4_ development in Bienertia, we performed transcriptome analysis of leaf tissues at three different developmental stages: young, intermediate, and mature (Koteyeva et al., 2016) (**Figure S1**). These different stages were classified based on the type and morphology of chloroplasts, and the development of the central vacuole (Park et al., 2009; Koteyeva et al., 2016). Total RNA extracted from leaf tissues at the three stages was subjected to RNA sequencing (RNA-seq) using the Illumina system.

A total of 268.4 million clean reads were obtained, covering >99% of raw Illumina data and with 45–46% GC content (**Table S1**). Assembly of the RNA-seq data yielded a total of 36,907 unigenes with N50 of 2,121 bp. Of the 36,907 unigenes, 18,581 (50.3% of the total), 25,943 (70.3%), and 21,592 (58.5%) unigenes were identified in the UniProt, non-redundant protein (nr) database of the National Center for Biotechnology Information (NCBI), and Araport11 database, respectively (**Table S2**). The assembly quality checker, Benchmarking Universal Single-Copy Orthologs (BUSCO), yielded 1,531 complete, 23 fragmented, and 60 missing genes at an E-value cut-off of 10^-3^ (**Figure 1A**; **Table S2**). Re-mapping of filtered transcripts using Bowtie2 resulted in 94.03–95.80% for all nine libraries prepared from the three developmental stages in three biological replicates (**Table S2**). BLAST search of the Bienertia transcriptome data against the nr database identified the following species as the three top hits in descending order: *Beta vulgaris* (9,533 unigenes, 25.8% of total unigenes), *Chenopodium quinoa* (8,906 unigenes, 24.1%), and *Spinacia oleracea* (5,323 unigenes, 14.4%) (**Dataset S2**).

First to examine whether the RNA samples prepared in this study accurately represented the developmental stages of Bienertia leaves, we performed classical multidimensional scaling (cMDS) plot analysis. Pairwise comparison of gene expression patterns was performed among the RNA-seq data of nine libraries. The results showed sufficient difference in gene expression patterns (Leading logFC dim 1) among leaf samples at different developmental stages (**Figure 1B**).

Next, we analyzed the genes differentially expressed between young and mature leaf samples (Young vs. Mature), based on log2-based fold change (Log2FC) at a false discovery rate (FDR) of < 0.05. A total of 3,965 upregulated and 2,674 downregulated differentially expressed genes (DEGs) were identified. To classify the DEGs according to their dynamic expression patterns during leaf development, we performed K-means clustering (**Figures 1C, D**). A total of 3,028 genes were identified that passed the 20.1 RS threshold as the minimum difference of DEGs set by NOISeq program. These genes were clustered into a total of 12 different K-means expression profiles (Cluster 1–12) in the heatmap (**Figures 1C, D**). The number of genes in these clusters varied from 60 to 552 (**Figure 1D**). The expression profiles of genes in Clusters 1, 2, 4 and 10 showed an inverse relationship with those of genes in Clusters 9, 7, 8 and 12, respectively (**Figures 1C, D**). Clusters 1 and 9 contained genes that showed an increase in the average expression level during leaf development; Clusters 5 and 6 included genes that were upregulated at the intermediate stage of development; and Clusters 1, 9, and 10 comprised genes upregulated at the mature stage (**Figure 1D**). The average Log2FC values of DEGs identified in the Young vs Mature comparison increased in the following order: 0.15 (Cluster 6), 0.71 (Cluster 5), 1.22 (Cluster 9), 1.36 (Cluster 1), and 1.43 (Cluster 10). To validate DEGs identified in the RNA-seq dataset, we examined the expression pattern of 12 genes at different leaf developmental stages by quantitative real-time PCR (qRT-PCR). The expression patterns determined by qRT-PCR matched those obtained by RNA-seq for all 12 genes (**Figure 1E**).

To define the biological processes represented by the DEGs, we performed the Gene Ontology (GO) enrichment analysis for the K-means clusters. We found GO terms such as ‘Photosynthesis (GO:0015979)’, ‘Photosynthesis, light reaction (GO:0019684)’, ‘Tetrapyrrole metabolic process (GO:0033013)’, and ‘Photosystem II assembly (GO:0010207)’ in Cluster 1 (**Figure S2A**), and ‘Chloroplast relocation (GO:0009902)’, ‘Establishment of plastid localization (GO:0051667)’, and ‘Photoprotection (GO:0010117)’ in Cluster 9 (**Figure S2B**). ‘Plastid organization (GO:0009657)’ appeared in both Clusters 1 and 9. These results suggest that genes in Clusters 1 and 9 are involved in photosynthetic energy production (GO:0015979, GO:0019684, and GO:0010117) and development of chloroplasts (GO:0033013, GO:0010207, GO:0009902, GO:0051667, and GO:0009657) (**Figures S2A, B**). Cluster 10, which also represented genes upregulated at the mature stage, included the following GO terms: GO:0015979, GO:0019684, GO:0010207, and GO:0009657 (**Figure S2C**). Clusters 5 and 6 represented genes upregulated only at the intermediate stage; however, these two clusters differed from each with respect to the GO terms (**Figures S2D, E**). Cluster 5 included GO terms such as ‘Pigment biosynthetic process (GO:0046148)’, ‘Thylakoid membrane organization (GO:0010027)’, ‘Protein targeting to chloroplast (GO:0045036)’, ‘Cellular homeostasis (GO:0019725)’, and ‘Cell redox homeostasis (GO:0045454)’, which are related to the development and homeostasis of chloroplasts (**Figure S2D**), whereas Cluster 6 included GO terms such as ‘Oxidative phosphorylation (GO:0006119)’, ‘Cellular respiration (GO:0045333)’, and ‘Energy derivation by oxidation of organic compounds (GO:0015980)’, which are related to mitochondrial energy metabolism (**Figure S2E**). Only Cluster 3 contained GO terms such as ‘Photosynthesis’ (GO:0015979), ‘Photosynthesis, light reaction’ (GO:0019684), ‘Oxidative phosphorylation’ (GO:0006119), ‘Cellular respiration’ (GO:0045333), and ‘Energy derivation by oxidation of organic compounds’ (GO:0015980), which are related to energy metabolism-related processes in both chloroplasts and mitochondria (**Figure S2F**). The GO term ‘Generation of precursor metabolites and energy’ (GO:0006091) was common to five clusters (Clusters 1, 3, 6, 9, and 10) (**Figure S2**).

### Co-expression of energy metabolism and C_4_ photosynthesis-related genes in *Bienertia sinuspersici*

**Figure 2:**
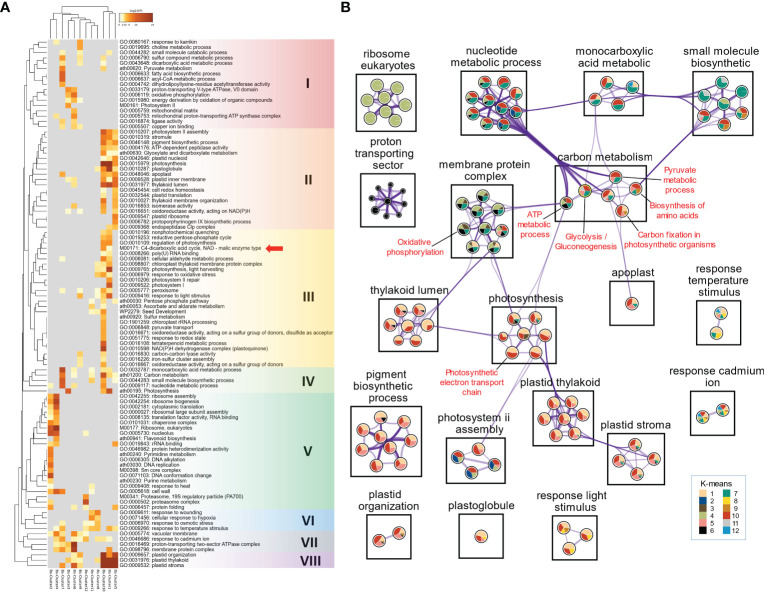
Functional enrichment analysis of DEGs using Metascape. **(A)** Heatmap of all statistically enriched functional categories (GO terms and KEGG/WP pathways) with hierarchical clustering (Group I to VIII). Accumulative hypergeometric p-values (-log10[P]) were used for filtering, and the remaining significant functional categories were hierarchically clustered into a tree, based on kappa similarity (*κ*). The *κ* value of 0.3 was applied as the threshold to cast the tree into category clusters. Red arrow indicates the ‘C_4_-dicarboxylic acid cycle, NAD-malic enzyme type’ (M00171) pathway. **(B)** GO/KEGG/WP semantic network marked by K-means clusters. Color ratio in each circular sector represents K-means cluster of genes within functional category (node). Purple lines (edges) indicate *κ* > 0.3 between nodes, and thickness of edges indicates the strength of the relationship between functional categories.

To better understand the correlation between gene expression patterns and biological functions, we performed GO term and Kyoto Encyclopedia of Genes and Genomes (KEGG) and WikiPathways (WP) pathway enrichment analyses of DEGs in each cluster (**Figure 2A**). Top 100 enriched GO terms and KEGG/WP pathways for the K-means clusters were hierarchically organized and represented in a heatmap (**Figure 2A**). We found that GO terms and KEGG/WP pathways were clustered into eight groups (I–VIII) based on gene expression profiles (**Figure 2A**). The ‘C_4_-dicarboxylic acid cycle, NAD-malic enzyme type’ (M00171) pathway clustered close to GO terms such as ‘Regulation of photosynthesis’ (GO:0010109), ‘Reductive pentose-phosphate cycle’ (GO:0019253), ‘Nonphotochemical quenching’ (GO:0010196), and ‘Poly(U) RNA binding’ (GO:0008266), which were also found in Clusters 1 and 10 along with all SCC_4_ genes (**Figure 2A**), indicating that these biological activities are closely connected to the NAD-ME-type C_4_ photosynthesis in Bienertia. A previous study showed that PCs in Bienertia display high CEF activity (Offermann et al., 2015). GO terms such as ‘Photosystem I’ (GO:0009522) and ‘NAD(P)H dehydrogenase complex (plastoquinone)’ (GO:0010598) also clustered together with ‘C_4_-dicarboxylic acid cycle, NAD-malic enzyme type’ (M00171) in the same group (Group III) (**Figure 2A**). These results suggest that genes related to CEF in PCs were coexpressed with those related to C_4_ photosynthesis in Bienertia. By contrast, mitochondrial energy metabolism-related terms such as ‘Pyruvate metabolism’ (ath00620), ‘Oxidative phosphorylation’ (GO:0006119), and ‘Mitochondrial proton-transporting ATP synthase complex’ (GO:0005753) clustered together with ‘Photosystem II’ (M00161) in Group I, which also contained the GO terms ‘Dicarboxylic acid metabolic process’ (GO:0043648) and ‘Energy derivation by oxidation of organic compounds’ (GO:0015980) (**Figure 2A**). Thus, these results indicate that the oxidation of organic compounds (which results in the production of tricarboxylic acid [TCA] cycle metabolites and C_4_ cycle metabolites) is closely related to mitochondrial energy metabolism and PSII. Furthermore, these results raise the possibility that the SCC_4_ system is established neither by a single regulon for the activity of PCs, nor by the simple integration of energy-generating systems in both chloroplasts and mitochondria.

Next, we performed semantic network analysis of enriched functional categories (WP/KEGG/WP) to elucidate the pathways underlying carbon metabolism in Bienertia. This analysis categorized the entire cellular functional categories into 12 groups, such as carbon metabolism, membrane protein complex, photosynthesis, plastid thylakoid, etc. Each of these groups contained 1–10 nodes (functional categories) within each individual group. Each functional category in the 12 groups also contained circular sectors on the information of K-means cluster. Functional categories with kappa similarity (*κ*) > 0.3 were represented as edges (connecting lines) to indicate the similarity in biological activity (**Figure 2B**). The ‘Carbon metabolism’ group in Bienertia included the functional categories of ‘ATP metabolic process’ (GO:0046034), ‘Glycolysis/Gluconeogenesis’ (ko00010), ‘Carbon fixation in photosynthetic organisms’ (ath00710), ‘Biosynthesis of amino acids’ (ath01230), and ‘Pyruvate metabolic process’ (GO:0006090). As indicated by *κ* and the DEGs represented in Clusters 6, 7 and 10, functional categories such as ‘ATP metabolic process’, ‘Glycolysis/Gluconeogenesis’, and ‘Pyruvate metabolic process’ in the ‘Carbon metabolism’ group had a strong connection as indicated by the large number of edges with functional categories such as ‘Nucleobase-containing small molecule metabolic process’ (GO:0055086), ‘Organophosphate metabolic process’ (GO:0019637), ‘Purine nucleotide metabolic process’ (GO:0006163), ‘Purine-containing compound metabolic process’ (GO:0072521), ‘Purine ribonucleotide metabolic process’ (GO:0009150), ‘Nucleoside phosphate metabolic process’ (GO:0006753)’, ‘Ribose phosphate metabolic process’ (GO:0019693), and ‘Ribonucleotide metabolic process’ (GO:0009259) in the ‘Nucleotide metabolic process’ group. Functional categories including ‘Cellular amino acid metabolic process’ (GO:0006520) and ‘Alpha-amino acid metabolic process’ (GO:1901605) in the ‘small molecule biosynthetic’ group also showed a strong relationship with the functional category of ‘Biosynthesis of amino acids’ in the ‘Carbon metabolism’ group, according to *κ* and the DEGs in Clusters 5, 7, 9, and 10. The functional categories of ‘Mitochondrial envelope’ (GO:0005740), ‘Mitochondrial protein-containing complex’ (GO:0098798), ‘Inner mitochondrial membrane protein complex’ (GO:0098800), and ‘Oxidative phosphorylation’ (ath00190) in the ‘Membrane protein complex’ group, and those of ‘Electron transport chain’ (GO:0022900) and ‘Generation of precursor metabolites and energy’ (GO:0006091) in the ‘Photosynthesis’ group showed a correlation with ‘ATP metabolic process’ in the ‘Carbon metabolism’ group, according to *κ* and the DEGs in Clusters 3, 4, 6, and 7. Moreover, DEGs related to mitochondrial/chloroplastic electron transport, namely those in the functional categories of ‘Oxidative phosphorylation’ (ath00190) and ‘Photosynthetic electron transport chain’ (GO:0009767) in the ‘Membrane protein complex’ group and ‘Photosynthesis’ group, respectively, were coexpressed with the DEGs related to ‘ATP metabolic process’ in the ‘Carbon metabolism’ group, displayed by Clusters 3, 4, 6, 7, and 10 (**Figure 2B**). However, a few genes represented by Cluster 10 in the ‘ATP metabolic process’ did not show a connection with the ‘Membrane protein complex’ group. This suggests that the ‘ATP metabolic process’ does not play a major role in biological processes related to the mitochondrial membrane protein complex at the mature stage.

The chloroplast-related groups such as ‘Thylakoid lumen’, ‘Photosynthesis’, ‘Plastid thylakoid’, ‘Plastid stroma’, etc. were represented mostly by Clusters 1, 5, and 10. On the other hand, metabolism-related groups such as ‘Nucleotide metabolic process’, ‘Monocarboxylic acid metabolic’, and ‘Small molecule biosynthetic’ were represented by Clusters 7 and 10 (**Figure 2B**). Clusters 1, 5, 9, and 10 contained upregulated DEGs involved in the functional category of ‘Carbon fixation in photosynthetic organisms’, which contained the ‘C_4_-dicarboxylic acid cycle, NAD-malic enzyme type’ (M00171) pathway. By contrast, functional categories such as ‘Biosynthesis of amino acids’, ‘Glycolysis/Gluconeogenesis’, and ‘Pyruvate metabolic process’, which constitutes the ‘carbon metabolism’ pathway, represented DEGs in Clusters 10 (upregulated genes) and 7 (downregulated genes) (**Figure 2B**). Thus, one possibility is that the genes in these three pathways might have been recruited to establish the C_4_ metabolic pathway via alterations in their expression patterns during the evolution of the SCC_4_ system in Bienertia.

### A gene related to high-light acclimation exhibits SCC_4_-specific expression in *Bienertia sinuspersici* and *Suaeda aralocaspica*

**Figure 3:**
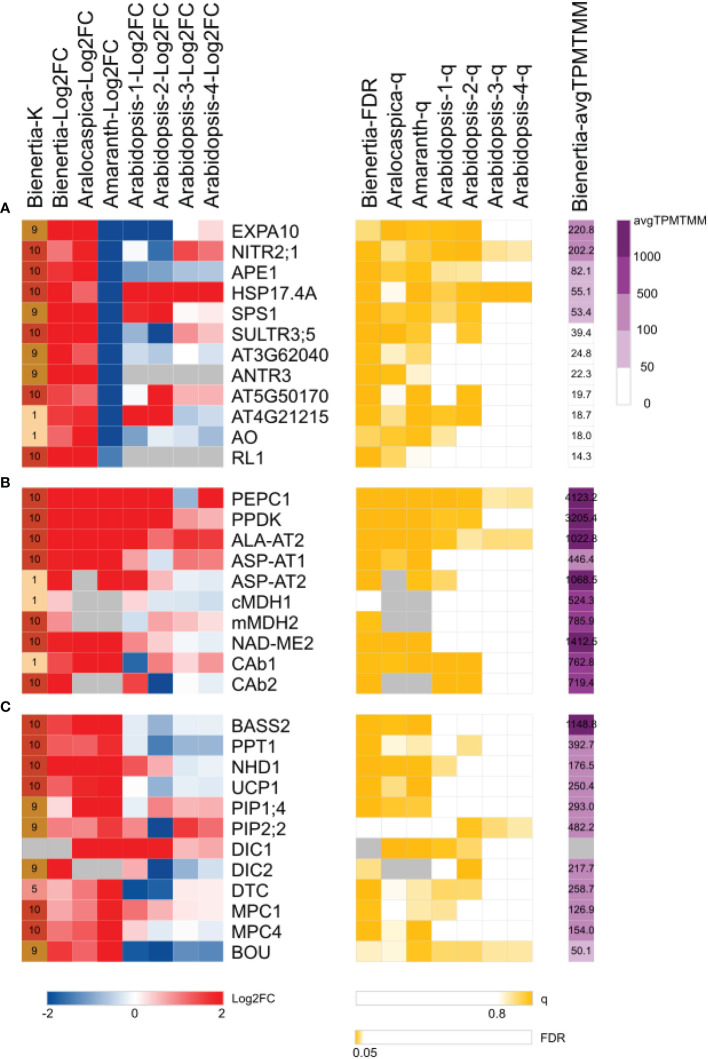
Heatmap of Bienertia genes exhibiting **(A)** single-cell C_4_-specific expression, **(B)** NAD-ME type C_4_ metabolic enzymes, and **(C)** NAD-ME type C_4_ metabolic transporters. Change in gene expression levels (left) is represented by log2 fold change (Log2FC). Red and blue colors indicate upregulated and downregulated genes, respectively. The significance of Log2FC (middle; yellow) is represented by FDR< 0.05 (EdgeR) or q > 0.8 (NOISeq). Purple color (right) indicates the gene expression level (avgTPMTMM). Gray color represents no data (NA). X-axis represents K-means clusters (K) or Log2FC of samples: Bienertia (*Bienertia sinuspersici*, Young vs Mature), Aralocaspica (*Suaeda aralocaspica*, Young vs Mature), Amaranth (*Amaranthus hypochondriacus*, Young vs Mature), Arabidopsis-1 (*Arabidopsis thaliana*, Young vs Mature, CT-aCT), Arabidopsis-2 (*Arabidopsis thaliana*, Young vs Mature, CTSAM-SCLF), Arabidopsis-3 (*Arabidopsis thaliana*, Non-stress vs Stress, RC-RH), Arabidopsis-4 (*Arabidopsis thaliana*, Non-stress vs Stress, RC-RSH). Y-axis represents gene names or Araport11 identifiers. Detailed information of abbreviations is provided in **Dataset S3** and **S4**.

We asked how similar or different the SCC_4_ system of Bienertia is from that of other SCC_4_ or Kranz anatomy C_4_ systems. Using specific criteria for SCC_4_ species (Log2FC > 1, FDR < 0.05, q > 0.8) and Kranz anatomy C_4_ species (Log2FC < -1, q > 0.8), we identified a few genes showing different expression patterns between SCC_4_ species (*Bienertia sinuspersici* [Bienertia] and *Suaeda aralocaspica* [Aralocaspica]) and Kranz anatomy C_4_ species (*Amaranthus hypochondriacus* [Amaranth]). These genes included Expansin-A10 (*EXPA10*; AT1G26770), Chloroplastic nitrite transporter (*NITR2;1*; AT5G62720), and Acclimation of photosynthesis to environment 1 (*APE1*; AT5G38660), which were upregulated at the mature stage only in SCC_4_ species (**Figure 3A**). According to previous studies, NITR2;1 plays a role in the nitrogen assimilation pathway (Maeda et al., 2014), and APE1 regulates high light acclimation to stabilize PSII via unstacking the thylakoids. The expression pattern of *APE1* in this study is consistent with the proposed role of APE1 in the green alga *Chlamydomonas reinhardtii* and in land plants (Walters et al., 2003; Chazaux et al., 2020; Trösch et al., 2022). This led us to speculate that APE1 plays a role in the production of different types of thylakoid stacks in PCs and CCs in Bienertia (see Discussion).

### Genes encoding NAD-ME type C_4_ enzymes are upregulated during leaf maturation

We further analyzed the transcriptome data to get additional clues regarding the establishment of C_4_ photosynthesis from the DEGs identified at the three developmental stages. One hypothesis is that the SCC_4_ system in Bienertia evolved from the C_3_ photosynthetic system during leaf maturation (Lara et al., 2008; Park et al., 2009; Galili et al., 2014; Koteyeva et al., 2016). Another hypothesis is that C_4_ metabolic genes were derived from those involved in the metabolism of amino acids and organic acids in C_3_ plants (Aubry et al., 2011; Igamberdiev & Eprintsev, 2016; Ludwig, 2016; Rao & Dixon, 2016; Rao et al., 2016; Blätke & Bräutigam, 2019; Khoshravesh et al., 2020; Borghi et al., 2022). Consistent with these hypotheses, several genes involved in the metabolism of amino acids and organic acids (carboxylates) were upregulated at the mature stage and were represented in Clusters 1 and 10 (**Figure 3B**). Cluster 1 contained two cytosolic C_4_ enzyme-encoding genes including aspartate aminotransferase 2 (*ASP-AT2*) and beta carbonic anhydrase 1 (*CAb1*), and Cluster 10 included genes such as pyruvate, phosphate dikinase (*PPDK*), phosphoenolpyruvate carboxylase 1 (*PEPC1*), aspartate aminotransferase 1 (*ASP-AT1*), alanine aminotransferase 2 (*ALA-AT2*), beta carbonic anhydrase 2 (*CAb2*), mitochondrial malate dehydrogenase 2 (*mMDH2*), and NAD-dependent malic enzyme 2 (*NAD-ME2*) (**Figure 3B**). These NAD-ME type C_4_ genes, which regulate the interconversion of the dicarboxylate and monocarboxylate groups of carboxylic acids through decarboxylation and carboxylation, respectively, were highly upregulated or expressed at the mature stage.

Next, we compared the Young vs Mature transcriptome data of four plant species, Bienertia (single/NAD-ME type C_4_ plant), Aralocaspica (single/NAD-ME type C_4_ plant), Amaranth (Kranz/NAD-ME type C_4_ plant), and Arabidopsis (C_3_ plant), to compare the expression patterns of C_4_ genes. We found that the NAD-ME type C_4_ photosynthetic genes were highly upregulated at the mature stage compared with the young stage in all three NAD-ME type C_4_ plants, regardless of the C_4_ type (SCC_4_ or Kranz) (**Figure 3B**). Among these C_4_ related genes, *ALA-AT2*, *PEPC1*, and *PPDK* were also highly upregulated in Arabidopsis, a C_3_ plant (**Figure 3B**), supporting the idea that some C_4_ genes have been derived from those involved in C_3_ photosynthesis. In fact, *ALA-AT2*, *PEPC1*, and *PPDK* were the top three genes with the highest Log2FC values (2.85, 2.83, and 2.55, respectively). By contrast, *PEPC1*, *PPDK*, and *NAD-ME2* were the top three genes with the highest transcript levels (average of Transcript Per Million normalized by Trimmed Mean of the M values [avgTPMTMM] = 4123.15, 3205.42, and 1412.48, respectively).

We aimed to assemble the SCC_4_ pathway by incorporating carbon metabolic flows based on highly expressed genes (avgTPMTMM > 50) and pathway databases (e.g., BioCyc and Rhea). We gathered a compilation of genes involved in various metabolic pathways in both C_3_ and C_4_ species. Our focus was on mitochondrial/cytosolic SCC_4_ genes that play a critical role in the NAD-ME type C_4_ pathway in C_4_ species, as well as in anaplerotic reactions in the TCA cycle and malate valve in C_3_ species (Aubry et al., 2011; Lambers et al., 2015; Igamberdiev and Eprintsev, 2016; Gakière et al., 2018; Selinski and Scheibe, 2019; Lim et al., 2020; Selinski and Scheibe, 2021; Sweetlove et al., 2010). In addition, we analyzed the chloroplastic SCC_4_ gene, PPDK, which serves as a vital component in supplying phosphoenolpyruvate (PEP) to the shikimate pathway for lignin biosynthesis in C_3_ species. Moreover, PPDK is responsible for maintaining carbon/nitrogen balance and aiding in gluconeogenesis for the synthesis of starch and sucrose in maize (Hibberd and Quick, 2002; Aubry et al., 2011; Tcherkez et al., 2011; Zhang et al., 2018).

### Comparative transcriptomic analysis of SCC_4_ cycle transporter genes among single-cell/Kranz and C_3_/C_4_ metabolic type species

In the NAD-ME type C_4_ cycle, C_4_ metabolites cross the membranes of chloroplasts and mitochondria. Hence, to better understand the SCC_4_ cycle of Bienertia, we identified C_4_ metabolite transporters. Comparative transcriptome analysis of four species included in this study revealed that genes encoding NAD-ME type C_4_ metabolic enzymes and NAD-ME type C_4_ metabolite transporters were highly upregulated (Log2FC = 0.55–2.76) or highly expressed (avgTPMTMM = 176.51–1148.81) (**Figures 3B, C**). These included genes encoding chloroplastic transporters such as phosphoenolpyruvate/phosphate translocator 1 (PPT1), sodium/pyruvate cotransporter BILE ACID: SODIUM SYMPORTER FAMILY PROTEIN 2 (BASS2), and sodium/proton antiporter 1 (NHD1) (Furumoto et al., 2011; Offermann et al., 2015; Schlüter et al., 2016), and mitochondrial transporters such as dicarboxylate/tricarboxylate transporter (DTC) and dicarboxylate carrier (DIC) (Offermann et al., 2015; Schlüter et al., 2016).

**Figure 4:**
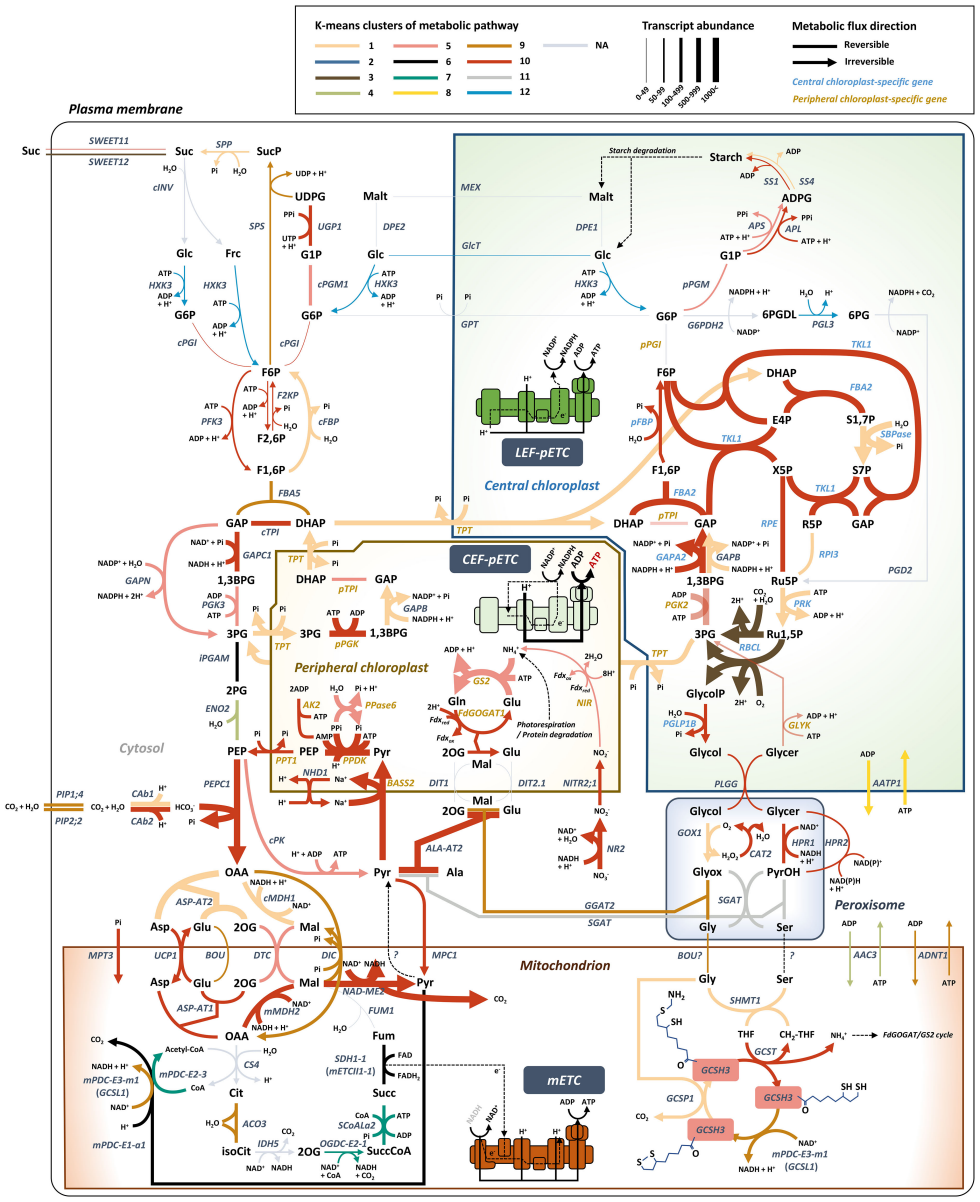
Model depicting central carbon metabolism in Bienertia marked by K-means cluster, transcript abundance, and reversible/irreversible enzyme bioinformatic information. The most probable unigene is selected for the representative metabolic pathway model. Detailed information of abbreviations is provided in **Dataset S6**.

To identify new transporters potentially involved in SCC_4_ metabolite transport, we compared our transcriptome data with the transporters in TCDB and UniProt (**Dataset S5**). Uncoupling protein 1 (*UCP1*) was upregulated at the mature stage (Log2FC = 1.24) and its transcript was abundant (avgTPMTMM = 250.40) (**Figure 3C**, **4**). UCP1 is involved in aspartate influx into and glutamate efflux from mitochondria, which are important for the decarboxylation of C_4_ metabolites by mMDH and NAD-ME during photorespiration and the C_4_ cycle (Monné et al., 2018).

Mitochondrial pyruvate carrier 1 and 4 (MPC1/4) complex in mitochondria is known to play a role in pyruvate metabolism as a transporter by supplying pyruvate to the TCA cycle in C_3_ species. In addition, the MPC1/4 complex can also be considered as a bidirectional transporter in mitochondria for ALA-AT in the NAD-ME type C_4_ cycle (Halestrap, 1975; Gray et al., 2014). However, a recent study showed that the *mpc1* mutant Arabidopsis plants are able to export pyruvate from mitochondria to the cytosol, indicating the presence of an additional functioning pyruvate exporter in mitochondria (Le et al., 2021). In our transcriptome analysis, *MPC1* and *MPC4* genes grouped in Cluster 10; at the mature stage, these genes were upregulated (Log2FC = 0.68 and 1.07, respectively) with high transcript levels (avgTPMTMM = 126.95 and 154, respectively) (**Figure 3C**, **4**). However, the presence of mitochondrial pyruvate exporter was still not clear in Bienertia. It is possible that MPC activity is derived from some other cytosolic/mitochondrial NAD-ME type C_4_ genes that play a role at the TCA cycle in C_3_ plants.

Genes including plasma membrane intrinsic protein 1;4 (*PIP1;4*) (avgTPMTMM = 292.96) and plasma membrane intrinsic protein 2;2 (*PIP2;2*) (avgTPMTMM = 482.22), which grouped in Cluster 9 (**Figure 3C**, **4**), were expressed at high levels at the mature stage. These aquaporins are known to transport CO_2_ or water through the plasma membrane (Li et al., 2015; Rao and Dixon, 2016; Byrt et al., 2017). CO_2_ and water are used for carbon fixation via a process involving CAb1/2 and PEPC1 (Offermann et al., 2015; Rao and Dixon, 2016).

A BOUT DE SOUFFLE (BOU) transporter is related to the glycine dehydrogenase complex (GDC) and serine hydroxymethyltransferase (SHMT) involved in photorespiration. A recent study suggested that BOU functions as a glutamate transporter in photorespiration (Kuhnert et al., 2021). *BOU* was found in Cluster 9 and showed an inverse relationship with the expression pattern of *ASP-AT2* in Cluster 1. Thus, it is possible that BOU constitutes a shunt pathway for replenishing glutamate levels in mitochondria. By contrast, ASP-AT2, which is involved in the conversion of glutamate to 2-oxoglutarate, functions in the indirect import of glutamate into mitochondria. Similarly, oxaloacetate (OAA) is imported into mitochondria either directly by DIC or indirectly via its conversion into aspartate by ASP-AT2 (**Figure 4**).

The top three most highly expressed SCC_4_ transporter genes, namely, *BASS2*, *PPT1*, and *PIP2-2*, are related to the primary and regenerative steps of the C_4_ cycle, along with PEPC1 and PPDK, respectively. Moreover, PC-specific SCC_4_ transporters (*BASS2*, *PPT1*, and *NHD1*; avgTPMTMM = 572.69) showed higher transcript levels than mitochondrial SCC_4_ transporters (*UCP1*, *DTC*, *DIC*, *MPC1/4*, and *BOU*; avgTPMTMM = 201.55) (**Figure 4**).

### NAD-ME type C_4_ genes are closely related to mitochondrial redox regulation and chloroplastic electron transport chain

Analysis of DEGs identified in the Young vs. Mature comparison revealed that the transcript levels of malate dehydrogenases *cMDH1* and *mMDH2* showed low fold change (Log2FC = 0.42 and 0.85, respectively) (**Figure 3B**). These results were consistent with those of previous studies on Bienertia and Amaranth; in those studies, no significant change was observed in cMDH1 and mMDH2 protein levels during leaf maturation or in *cMDH1* transcript level under adverse environmental conditions (Offermann et al., 2015; Vera Hernández et al., 2018). This suggests that *MDHs* are expressed in a constitutive manner and their regulation is different from that of other C_4_ genes. Our transcriptomic results showed that C_4_ species exhibit high expression levels of *cMDH1* and *mMDH2* (avgTPMTMM = 524.27 and 785.87, respectively) (**Figure 3B**, **4**). These expression patterns of *cMDH1* and *mMDH2*, together with the high expression levels of *DTC* and *DIC* genes in C_4_ species, are in agreement with the indirect transport of NADH using malate valve enzymes (MDHs) and malate valve antiporters (DTC and DIC) reported in C_3_ species (Selinski and Scheibe, 2019).

The top five genes with the highest transcript abundance (avgTPMTMM) related to photosystem and carbon fixation included Chlorophyll a-b binding protein 1 (*LHCB1.3*; AT1G29930), Oxygen-evolving enhancer protein 1-2 (*PSBO2*; AT3G50820), Chlorophyll a-b binding protein CP26 (*LHCB5*; AT4G10340), Ribulose bisphosphate carboxylase small subunit 1A (*RBCS-1A*; AT1G67090), and *PEPC1* (AT1G53310) (**Dataset S1**). Previously, proteomic analysis revealed Ribulose bisphosphate carboxylase large chain (*rbcL*; ATCG00490), ATP synthase subunit beta (*atpB*; ATCG00480), *PPDK* (AT4G15530), *PEPC1* (AT1G53310), and ATP synthase subunit alpha (*atpA*; ATCG00120) as the top five most abundant proteins. They are also related to carbon fixation (Offermann et al., 2015). Among the genes related to the GO term ‘Photosynthesis’, Photosystem I P700 chlorophyll a apoprotein A2 (*PsaB*; ATCG00340), Photosystem I P700 chlorophyll a apoprotein A1 (*PsaA*; ATCG00350), *PEPC1* (AT1G53310), Cytochrome b6 (*PetB*; ATCG00720), Early light-induced protein 1 (*ELIP1*; AT3G22840), *PPDK* (AT4G15530), Photosynthetic NADH dehydrogenase (NDH) subunit of lumenal location 3 (*PNSL3*; AT3G01440), Chaperonin-like RbcX protein 2 (*RBCX2*; AT5G19855), and Photosystem I chlorophyll a-b binding protein 3-1 (*LHCA3*; AT1G61520) showed a significant increase in transcript levels (Log2FC > 2, FDR < 0.05). Of these nine genes, five genes (*PsaA*, *PsaB*, *PetB*, *LHCA3*, and *PNSL3*) were related to CEF around PSI, four genes (*PsaA*, *PsaB*, *PetB*, and *LHCA3*) were related to ‘mRNA binding’ (GO:0003729), and six genes (*PsaA*, *PetB*, *ELIP1*, *PNSL3*, *RBCX2*, and *LHCA3*) were related to ‘Chloroplast thylakoid membrane’ (GO:0009535) (**Dataset S1**). Thus, CEF around PSI appeared to be important for leaf development in Bienertia.

**Figure 5:**
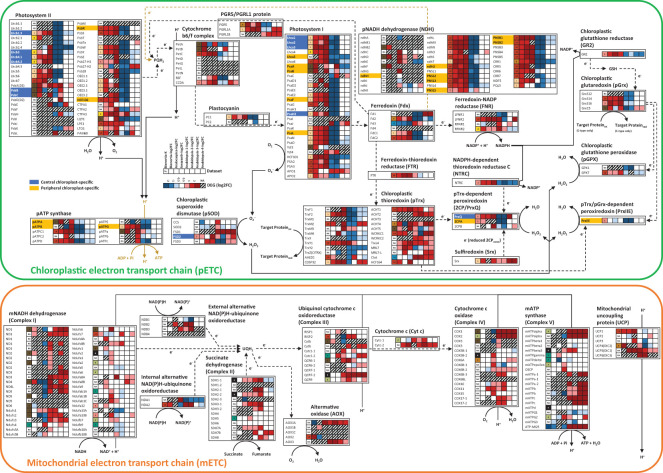
The electron transport chain pathway of chloroplasts and mitochondria in Bienertia. Solid lines, metabolic flows; dotted lines, electron flows; blue color, central chloroplast (CC)-specific localization; yellow color, peripheral chloroplast (PC)-specific localization. Upregulated and downregulated genes are marked in red and blue, respectively, based on the Log2FC data. No data (NA) is indicated in gray. X-axis of the heatmap represents K-means clusters (K) or Log2FC of samples: Bienertia (*Bienertia sinuspersici*, Young vs Mature), Aralocaspica (*Suaeda aralocaspica*, Young vs Mature), Amaranth (*Amaranthus hypochondriacus*, Young vs Mature), Arabidopsis-1 (*Arabidopsis thaliana*, Young vs Mature, CT-aCT), Arabidopsis-2 (*Arabidopsis thaliana*, Young vs Mature, CTSAM-SCLF), Arabidopsis-3 (*Arabidopsis thaliana*, Non-stress vs Stress, RC-RH), Arabidopsis-4 (*Arabidopsis thaliana*, Non-stress vs Stress, RC-RSH); Y-axis of the heatmap represents genes. The details of abbreviations are provided in **Dataset S7**.

Antioxidant genes involved in the protection of chloroplasts from high light intensity were also coexpressed with genes involved in photosynthesis-related systems such as the redox system (**Figure 5**). Genes involved in the chloroplastic electron transport chain (pETC) were upregulated at the mature stage of leaf development in all three NAD-ME type C_4_ plants including Bienertia, Aralocaspica, and Amaranth. By contrast, genes involved in the mitochondrial electron transport chain (mETC) were not upregulated in the mature leaves of these three plants but were in those of Arabidopsis, a C_3_ plant (**Figure 5**).

## Discussion

The most prominent feature of Bienertia is dimorphic chloroplasts, which develop along with leaf maturation. CCs display typical stacked thylakoids and grana as in C_3_ chloroplasts, whereas PCs exhibit minimal thylakoid stacking with low levels of PSII (Mai et al., 2019). However, the process of dimorphic chloroplast development in the mature leaf cells of Bienertia remains poorly understood. One hypothesis was that the dimorphic chloroplasts are developed by preventing PC-specific proteins from being targeted to CC, but not by any novel sequences that are specific to PC-specific proteins (Offermann et al., 2015; Wimmer et al., 2017). However, other factors involved in the specific targeting are yet to be discovered. Our analysis of gene expression patterns revealed that certain genes such as TIC55 and TIC20-V encoding the components of the translocon at the inner envelope membrane of chloroplasts (TIC) were consistently upregulated in all C_4_ plant species. However, they did not show any clear correlation with dimorphic chloroplasts (see **Dataset S1**, **S5**). Thus, upregulation of certain TOC/TIC genes may potentially contribute to the development of dimorphic chloroplasts; it is not clear from the available data whether their expression patterns directly underlie the establishment of the SCC_4_ system or dimorphic organellar development. Our enrichment analysis revealed a high ranking for ‘Generation of precursor metabolites and energy’ in relation to ‘Photosynthesis’ and ‘Chloroplast organization’ during leaf development in Bienertia (see **Figure S2**). This finding led us to speculate that a few genes affecting energy metabolism, photosynthesis, and dimorphic chloroplast development might have contributes to the establishment of the SCC_4_ system.

One possible scenario is that biogenesis of PCs occurs through the suppression of thylakoid stacking; another possibility is that the low level of thylakoid stacks is a result of PSII disassembly. In this study, Clusters 1 and 10 represented the GO terms ‘Photosystem II assembly’, ‘Photosystem II repair’, and ‘Nonphotochemical quenching’ (**Figure 2**, **S2**). The co-occurrence of these GO terms raises the possibility that the disassembly of the PSII–light harvesting complex II (LHCII) supercomplex by Photosystem II 22 kDa protein (PsbS) and serine/threonine-protein kinase 7 (STN7) may contribute to the lower degree of thylakoid stacking in PCs because the process of PSII–LHCII disassembly mediated by PsbS and STN7 reduces the appressed regions of grana thylakoids during PSII repair and nonphotochemical quenching (NPQ) in response to light stress (Pribil et al., 2014; Dumas et al., 2016; Lu, 2016; Welc et al., 2021; Bielczynski et al., 2022; Wasilewska-Dębowska et al., 2022). Moreover, earlier studies on Arabidopsis and maize showed that PsbS and STN7 modulates the activity of PSII in such a way that thylakoid membranes become unstacked, leading to the activation of PSII-independent CEF. Thus, these proteins may be considered as factors potentially promoting the establishment of C_4_ photosynthesis (Pribil et al., 2014; Dumas et al., 2016; Wood and Johnson, 2020; Wasilewska-Dębowska et al., 2022). In this study, transcriptomic analysis revealed that *PsbS* and *STN7* genes were upregulated only in NAD-ME C_4_ species (**Dataset S1**). Accordingly, we considered that factors controlling PSII–LHCII disassembly under high light may be critical for the biogenesis of PC-type thylakoids and the induction of CEF in PCs. Moreover, we found that PSII stability-related genes encoding APE1 and peroxiredoxin Q (PrxQ) in Bienertia were upregulated during leaf maturation. APE1 can be a key factor affecting thylakoid stack modulation under high light condition in Bienertia (Chazaux et al., 2020; Trösch et al., 2022). In this study, the *APE1* gene showed single-cell specific upregulation in the mature leaves of Bienertia and Aralocaspica (**Figure 3A**). A recent study showed that APE1 plays a role in the photoprotection of PSII from light stress via the disassembly of the PSII–LHCII supercomplex; the *ape1* mutant of *C. reinhardtii* is unable to reduce thylakoid stacks during high light acclimation (Chazaux et al., 2020). On the other hand, antioxidant enzymes capable of scavenging hydrogen peroxide (H_2_O_2_), PrxQ and 2-Cys peroxiredoxin (2CP), differentially localize to CCs and PCs, respectively (Offermann et al., 2015). Our transcriptome analysis showed that genes encoding these peroxiredoxins were upregulated in Bienertia leaves at the mature stage (**Figure 5**). Unlike 2CP, however, PrxQ has been reported to bind to a PSII-binding protein on the thylakoid membrane to protect the PSII core against PSI-generated H_2_O_2_ (Lamkemeyer et al., 2006). Although interaction between APE1 and PrxQ is unknown, we speculate that the stabilization of the PSII–LHCII supercomplex in CCs may be a key feature under high light condition in Bienertia. The differential stacking of thylakoids in the two types of chloroplasts is closely related to their function; high levels of NDHs and low levels of PSII are required for CEF, which contributes to PPDK-catalyzed PEP biosynthesis from pyruvate in C_4_ photosynthesis. This is because excess ATP from CEF is used by PPDK (Offermann et al., 2015; Wasilewska-Dębowska et al., 2022). In this study, we found that genes involved in maintaining PSII stability and CEF were upregulated together with those involved in regulating SCC_4_ metabolism (**Figure 2**, **S2**). Hence, our transcriptomic data suggest that the expression of genes involved in SCC_4_ is related to that of genes involved in CEF in Bienertia.

In the dimorphic chloroplasts of Bienertia, triose-phosphate transporter (TPT) catalyzes a glycolytic shunt called ‘Triose-phosphate shuttle (TPS)’. During SCC_4_ photosynthesis, this shuttle can balance carbon sources (3PG and DHAP) and indirectly transfer reducing powers (ATP, NADH, and NADPH) between the cytosol and dimorphic chloroplasts (Facchinelli and Weber, 2011; Offermann et al., 2015). Our transcriptome data showed that the *TPT* gene was included in Cluster 1, which contained genes upregulated during leaf maturation (**Figure 4**). We, thus, considered a scenario that the C_4_ metabolite cycle between PCs and mitochondria is associated with altered pools of energy molecules from the CEF of PCs. However, the enrichment analysis of Bienertia DEGs revealed that biological activities of chloroplasts and mitochondria are mostly independent of each other in the gene expression patterns of K-means cluster, except for the genes involved in the C_4_ cycle (**Figure 2**). Moreover, mitochondrial NAD-ME type C_4_ enzymes require NADH, as in the case of mMDH, contrary to the ATP-dependent PPDK in PCs. Therefore, the mitochondrial SCC_4_ pathway is not directly related to ATP but to NADH. In plants, chloroplastic NADPH and mitochondrial NADH can be oxidized via the malate valve system, similar to the TPS system, to transfer the reducing powers to exochloroplastic and exomitochondrial locations, respectively (Taniguchi and Miyake, 2012; Driever and Kromdijk, 2013; Lim et al., 2020). In NADP-ME type C_4_ photosynthesis, the malate valve system of chloroplasts is closely related to the cell specialization of Kranz anatomy under high light intensity (Taniguchi and Miyake, 2012). It is possible that the Arabidopsis homologs of malate valve-related enzymes cMDH and mMDH and those of malate valve-related transporters DTC and DIC function in the SCC_4_ cycle in Bienertia. It is possible that cMDH1 plays a key role in the cycling of C_4_ metabolites between the cytosol and mitochondria in Bienertia. Conversion of mitochondrial OAA to malate and that of cytosolic malate to OAA in the malate valve may constitute the SCC_4_ cycle, and cytosolic NADH may be maintained at a high level for running the OAA cycle in Bienertia (**Figure 4**). On the other hand, the glycolysis leads to the production of PEP and pyruvate; PEPC1 and cMDH catalyze the reversible conversion of PEP to malate for replenishing the TCA cycle intermediates in the C_3_ system (Bandehagh and Taylor, 2020). Hence, we assessed whether cytosolic and mitochondrial SCC_4_ genes are closely related to the homeostasis of NADH and anaplerosis of TCA cycle intermediates in a single cell. Here, we assumed that the direction of certain reversible reactions in metabolic pathways can be controlled by the levels of reductants/oxidants (e.g., NADH/NAD^+^ and ATP/AMP). It has been suggested that high levels of ATP in PCs determine the direction of the chemical reaction mediated by PPDK. Therefore, we propose that the direction of a chemical reaction carried out by mMDH in the SCC_4_ pathway in the presence of high NADH level is the opposite to that carried out by mMDH in the TCA cycle within the mitochondria. A series of reactions involving NADH and ATP are required for exchanging pyruvate between mitochondria and chloroplasts to provide PEP, which serves as the CO_2_ acceptor in the PEPC1-mediated CO_2_ fixation reaction (**Figure 4**).

**Figure 6:**
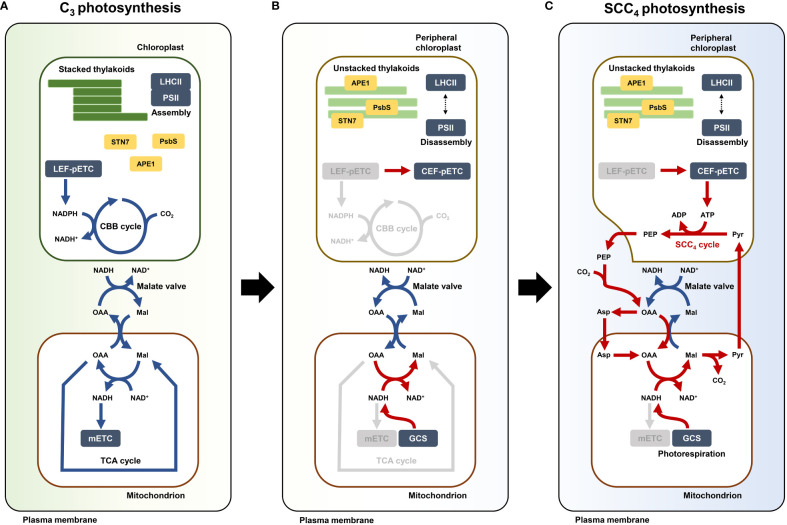
Model depicting establishment of single-cell C_4_ (SCC_4_) cycle in Bienertia. **(A)** C_3_ photosynthesis when CBB cycle and TCA cycle are operated by chloroplastic NADPH and mitochondrial NAD^+^, respectively. **(B)** Hypothetical transient state in photosynthesis by altered energy metabolism: electron flow change of pETC by disassembly of LHCII-PSII supercomplex and deactivated mETC. **(C)** Metabolic direction of SCC_4_ cycle (red arrows) established by supplies of peripheral chloroplastic ATP from CEF and mitochondrial NADH from photorespiratory GCS. CBB cycle (Calvin-Benson-Bassham cycle), TCA cycle (Tricarboxylic acid cycle), LHCII (Light harvesting complex II), PSII (Photosystem II core complex), LEF-pETC (Linear electron flow of chloroplastic electron transport chain [Photosystem]), CEF-pETC (Cyclic electron flow of chloroplastic electron transport chain [Photosystem]), mETC (Mitochondrial electron transport chain [Oxidative phosphorylation]), GCS (Glycine cleavage system), STN7 (Serine/threonine-protein kinase 7), PsbS (Photosystem II 22 kDa protein), APE1 (Acclimation of photosynthesis to environment 1), PEP (Phosphoenolpyruvate), Pyr (Pyruvate), Asp (Aspartate), OAA (Oxaloacetate), Mal (malate), NADPH (Reduced form of nicotinamide adenine dinucleotide phosphate), NADP^+^ (Oxidized form of nicotinamide adenine dinucleotide phosphate), NADH (Reduced form of nicotinamide adenine dinucleotide), NAD^+^ (Oxidized form of nicotinamide adenine dinucleotide), ATP (Adenosine triphosphate), ADP (Adenosine diphosphate), CO_2_ (Carbon dioxide).

Our transcriptome analysis revealed small changes in the expression of mETC genes in C_4_ species in comparison with Arabidopsis (**Figure 5**). A change in the local ATP and NADH levels in a cell can alter the direction of a carbon pathway (Liang et al., 2015; Lim et al., 2020). The altered Complex I mETC leads to different metabolic pools of amino acids and organic acids in the cytosol and mitochondria (Foyer et al., 2011). Arabidopsis *ndufs4* mutant plants exhibit a trace level of Complex I activity, and this defect leads to extensive metabolic changes in the TCA cycle, amino acid and organic acid metabolism, and redox metabolism (McCollum et al., 2019). Therefore, the low-level expression of mETC-related genes might have been a precondition for reorganizing pathways involving amino acids and organic acids. Subsequently, recycling of metabolites such as amino acids and organic acids through PEP synthesis by PPDK in the presence of excess ATP in PCs might have led to the establishment of a functional SCC_4_ cycle. Finally, the presence of excess NADH in mitochondria and excess ATP in PCs probably required genes involved in balancing the reducing powers between mitochondria and PCs within a cell, thereby reflecting the SCC_4_ cycle (**Figure 6**). We propose that altered energy metabolism of mitochondria is important for establishing the SCC_4_ system, including the formation of dimorphic chloroplasts within a cell.

In conclusion, we propose that the SCC_4_ system likely originated from the alteration of the malate valve in the mitochondrial NADH pool, and this change caused the production of C_4_ metabolites in the cytosol and mitochondria. In addition, further compensatory activity of PCs induces carboxylate circulation as C_4_ metabolite flux between PCs and mitochondria.

## Data availability statement

The datasets presented in this study can be found in online repositories. The names of the repository/repositories and accession number(s) can be found in the article/**Supplementary Material**.

## Author contributions

IH, W-YK, and JSK conceived the project. S-YH prepared RNA-seq sample. JSK provided RNA-seq data. S-YH assembled transcriptome and carried out computational analyses. S-YH and IH wrote the manuscript. All authors contributed to the article and approved the submitted version.
