# Supplementary material for: Comparative transcriptomics reveals the role of altered energy metabolism in the establishment of single-cell C4 photosynthesis in Bienertia sinuspersici
Source: Front Plant Sci. 2023 Jul 5;14:1202521. doi: 10.3389/fpls.2023.1202521 (PMC10354284; doi:10.3389/fpls.2023.1202521)
Supplement: Supplementary file 1 [file DataSheet_1.docx]

**Supplementary Information legends**

**Supplementary Figure 1.** Sampling of *Bienertia sinuspersici* leaf tissues for transcriptome analysis.

(A) Three stages of leaf development.

(B) Confocal microscopy z-stack images of chlorenchyma cells of Bienertia at the three developmental stages representing young, intermediate, and mature cells. The chlorophyll autofluorescent signal of chloroplasts was detected at a wavelength of 647 nm. Scale bar = 10 μm.

**Supplementary Figure 2.** Biological process subontology of GO (GO:BP) term enrichment analysis in each K-means cluster.

(A) Cluster 1, (B) Cluster 9, (C) Cluster 10, (D) Cluster 5, (E) Cluster 6, (F) Cluster 3. Dot color represents the adjusted p-value (-log10[FDR]). Dot size represents the number of DEGs in each GO term. X-axis indicates the DEGs of each GO term relative to the total gene number in each K-means cluster (GeneRatio).

**Supplementary Table 1.** Statistics of RNA-seq data of *Bienertia sinuspersici* generated in this study.

**Supplementary Table 2.** Metrics of *de novo* assembly of Bienertia transcriptome.

**Supplementary Table 3.** List of primers used to perform quantitative real-time PCR in this study.

**Supplementary Dataset 1.** Annotation of the transcriptome and differentially expressed genes (DEGs) of *Bienertia sinuspersici*.

**Supplementary Dataset 2.** Annotation of *Bienertia sinuspersici* based on the NCBI non-redundant protein **(**nr) database.

**Supplementary Dataset 3.** Details of genes showing SCC_4_-specific expression in *Bienertia sinuspersici* and *Suaeda aralocaspica*.

**Supplementary Dataset 4.** Details of genes encoding SCC_4_ enzymes and SCC_4_ transporters in *Bienertia sinuspersici*.

**Supplementary Dataset 5.** Annotation of transporter genes in *Bienertia sinuspersici*.

**Supplementary Dataset 6.** Details of abbreviations in model depicting central carbon metabolism in *Bienertia sinuspersici*.

**Supplementary Dataset 7.** Detailed information of the electron transport chain pathway in the chloroplasts and mitochondria of *Bienertia sinuspersici*.

**Supplementary Table 1.** Statistics of RNA-seq data of *Bienertia sinuspersici* generated in this study.

| **Sample** | **Sample type** | **Raw reads (paired)** | **Clean reads (paired)** | **%GC  of clean reads** | **Accession number (SRA)** |
| --- | --- | --- | --- | --- | --- |
| Y1 | Young leaf  (< 0.3 cm) | 34,729,178 | 34,660,184  (99.80%) | 45% | SRR22963771 |
| Y2 |  | 33,291,184 | 33,216,919  (99.77%) | 45% | SRR22963770 |
| Y3 |  | 33,620,307 | 33,546,741  (99.78%) | 46% | SRR22963769 |
| I1 | Intermediate leaf  (> 0.3 cm, < 0.7 cm) | 27,879,730 | 27,794,287  (99.69%) | 45% | SRR22963768 |
| I2 |  | 30,036,060 | 29,960,151  (99.74%) | 45% | SRR22963767 |
| I3 |  | 26,856,407 | 26,803,106  (99.80%) | 46% | SRR22963766 |
| M1 | Mature leaf  (> 2 cm, 1/3 tip) | 28,021,214 | 27,963,086  (99.79%) | 45% | SRR22963765 |
| M2 |  | 30,997,983 | 30,942,971  (99.82%) | 45% | SRR22963764 |
| M3 |  | 23,571,422 | 23,529,432  (99.82%) | 45% | SRR22963763 |

**Supplementary Table 2.** Metrics of *de novo* assembly of Bienertia transcriptome.

| **Metric** |  |
| --- | --- |
| Total contigs | 36,907 |
| Annotated transcripts by UniProt | 18,581 |
| Annotated transcripts by nr | 25,943 |
| Annotated transcripts by Araport11 | 21,592 |
| Contig N50 (bp) | 2,121 |
| Contig N90 (bp) | 816 |
| Maximum contig length (bp) | 35,033 |
| Minimum contig length (bp) | 183 |
| GC content overall (%) | 40 |
| Total nucleotides (bp) | 55,544,049 |
| Remapping rates of samples by Bowtie2 (%) | 94.03-95.80 |
| BUSCO complete (C) (score, %)† | 1531 (94.9) |
| BUSCO complete and single-copy (S) (score, %)† | 1047 (64.9) |
| BUSCO complete and multiple-copy (D) (score, %)† | 484 (30.0) |
| BUSCO fragmental (F) (score, %)† | 23 (1.4) |
| BUSCO missing (M) (score, %)† | 60 (3.7) |
| †BUSCO analysis was conducted using Embryophyta database and transcriptome mode with 1e^-3^ e-value cutoff | |

**Supplementary Table 3.** List of primers used to perform quantitative real-time PCR in this study.

| **Gene name** | **Primer sequence (5'-3')** | **Efficiency (%)** | ***R*^2^** | **Length (bp)** |
| --- | --- | --- | --- | --- |
| BsRPT6A | F: TGTACTGAAGCAGGTATGTTTGC | 100.84 | 0.9926 | 59 |
|  | R: TCTTCCTGTGTTACGTGAATCC |  |  |  |
| BsSAND | F: ACACTCCAACTAAGCACCTCC | 98.37 | 0.9830 | 97 |
|  | R: TCTTCGTGTTTGGGAATCGC |  |  |  |
| BsPPDK | F: GGGATTGGCTTTCTCTTAATGGA | 97.36 | 0.9982 | 68 |
|  | R: AGGTGGTGAAAGCGGTTCTTT |  |  |  |
| BsPEPC1 | F: CCCGCTTTGTTGAGTACTTCC | 94.59 | 0.9978 | 68 |
|  | R: GCTTCCGATATTCATGCGTCC |  |  |  |
| BsNAD-ME2 | F: ATTGGGTTGGGAGCTTTGC | 101.44 | 0.9997 | 82 |
|  | R: AGGAAGCAAGACATTCAGCG |  |  |  |
| BsCAb1 | F: TAAAGCCGGATCAAAAGCGGT | 101.89 | 0.9991 | 97 |
|  | R: CCCTTCCTTCATTCTCTTAATCGGA |  |  |  |
| BsCAb2 | F: ACGTGGCAGCTGCAAAGATA | 97.71 | 0.9705 | 87 |
|  | R: TGTCACTGGTACCGCCAATATA |  |  |  |
| BsASP-AT1 | F: AACGATCAACCTGCTCTGG | 101.62 | 0.9937 | 50 |
|  | R: GATTGGTATGTTCTGCTACAGTGG |  |  |  |
| BsASP-AT2 | F: CTTATCTGCTCCCATTGTTCC | 99.06 | 0.9985 | 71 |
|  | R: GTTCTTCTCCATGCTTGTGC |  |  |  |
| BscMDH1 | F: GTTGGTGGTTTCCCAAGG | 99.42 | 0.9975 | 58 |
|  | R: CATTCTTTGCCATCACATCC |  |  |  |
| BsmMDH2 | F: TCCCTCAAGTGCCTTTGC | 97.51 | 0.9986 | 55 |
|  | R: AGCTCAGGTTTCTGGTTATATGG |  |  |  |
| BsDTC | F: TCAGTTGGTCTTGGTGAAGC | 96.76 | 0.9995 | 56 |
|  | R: AATCCAGAAACGGCACTAGC |  |  |  |
| BsUCP1 | F: AACATCTCCGACGTGATCC | 98.37 | 0.9952 | 76 |
|  | R: GTTTGGTGGTCTAAGGATTGG |  |  |  |
| BsTPT | F: CCAACACCTTAGAGAGGGTAGC | 99.31 | 0.9989 | 55 |
|  | R: TTCAAGACGTTTCCAACTGC |  |  |  |
