# Supplementary figures and images for: Comparative transcriptomics reveals the role of altered energy metabolism in the establishment of single-cell C4 photosynthesis in Bienertia sinuspersici
Source: Front Plant Sci. 2023 Jul 5;14:1202521. doi: 10.3389/fpls.2023.1202521 (PMC10354284; doi:10.3389/fpls.2023.1202521)

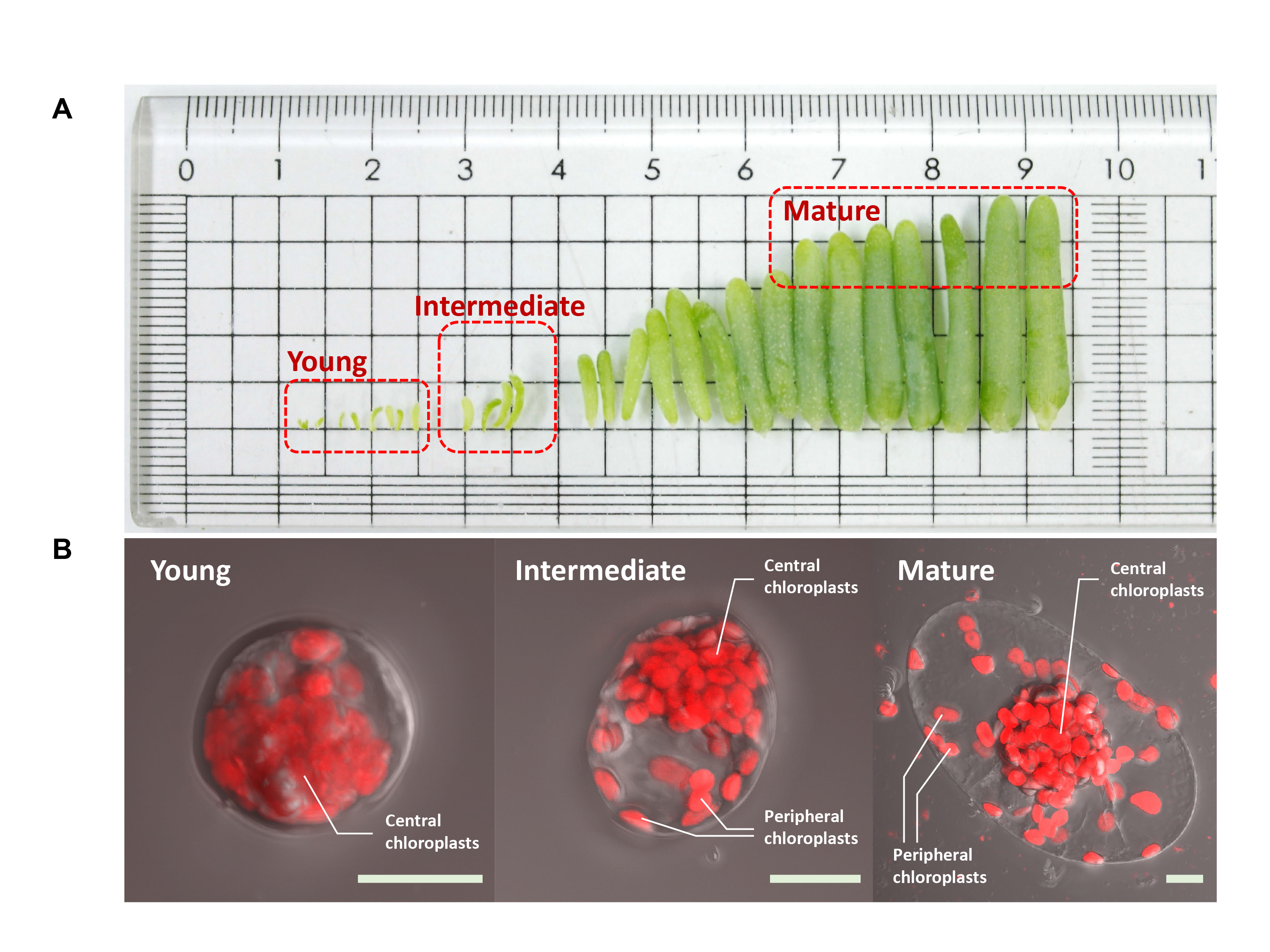

Supplement: Supplementary file 3 [file Image_1.tif]

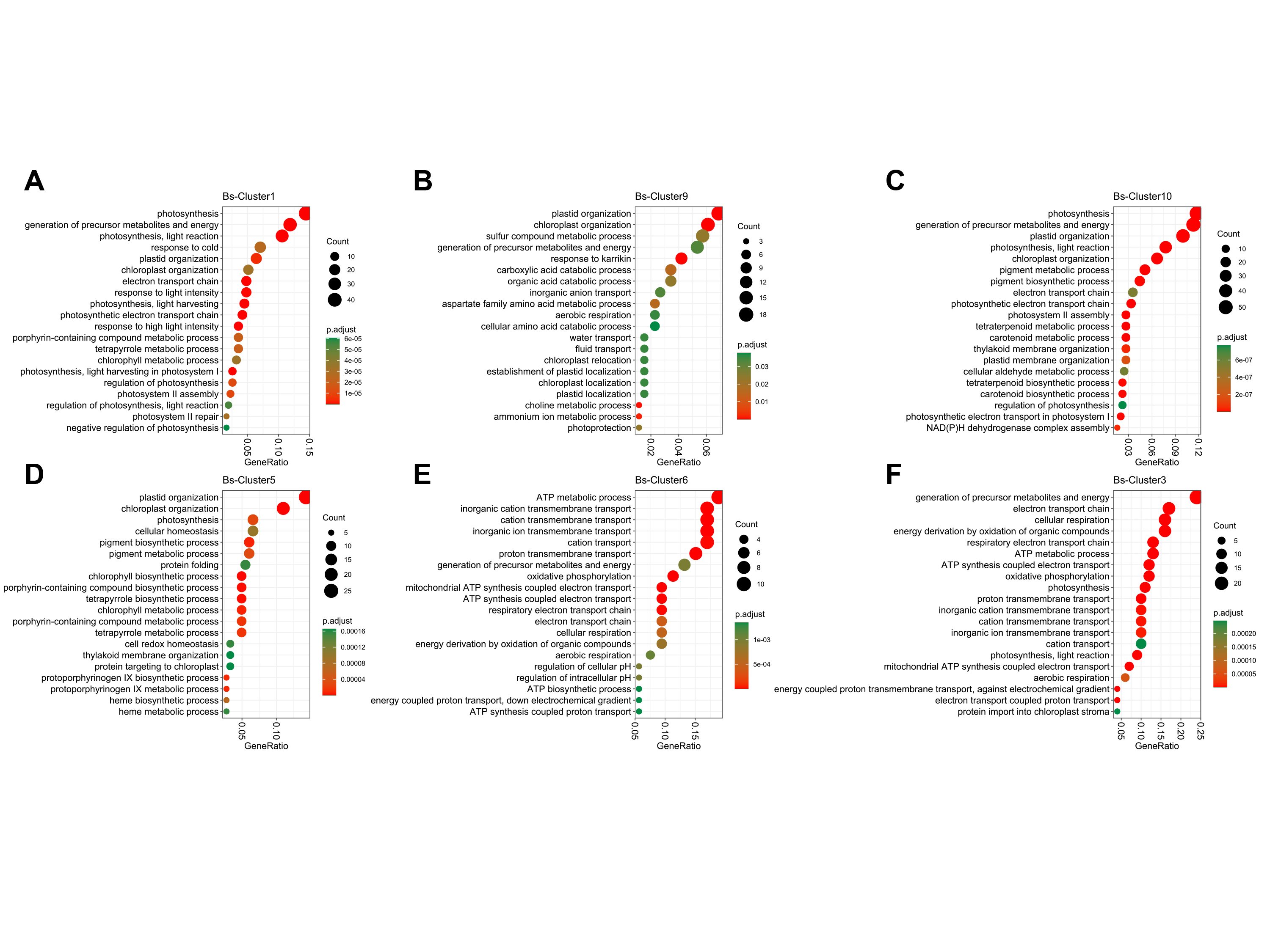

Supplement: Supplementary file 4 [file Image_2.tif]
